# Nutrition Knowledge Is Associated with Energy Availability and Carbohydrate Intake in Young Female Cross-Country Skiers

**DOI:** 10.3390/nu13061769

**Published:** 2021-05-22

**Authors:** Oona Kettunen, Maria Heikkilä, Vesa Linnamo, Johanna K. Ihalainen

**Affiliations:** 1 Sports Technology Unit Vuokatti, Faculty of Sport and Health Sciences, University of Jyväskylä, Kidekuja 2, 88610 Vuokatti, Finland; vesa.linnamo@jyu.fi; 2 Faculty of Sport and Health Sciences, University of Jyväskylä, Rautpohjankatu 8, 40014 Jyväskylä, Finland; johanna.k.ihalainen@jyu.fi; 3 Department of Food and Nutrition, Faculty of Agriculture and Forestry, University of Helsinki, P.O. Box 66, 00014 Helsinki, Finland; mariaelisa.heikkila@gmail.com

**Keywords:** endurance athlete, macronutrient, periodized nutrition, protein, sports nutrition, winter sport

## Abstract

The aim of this study was to provide information on energy availability (EA), macronutrient intake, nutritional periodization practices, and nutrition knowledge in young female cross-country skiers. A total of 19 skiers filled in weighted food and training logs before and during a training camp. Nutrition knowledge was assessed via a validated questionnaire. EA was optimal in 11% of athletes at home (mean 33.7 ± 9.6 kcal·kgFFM^−1^·d^−1^) and in 42% at camp (mean 40.3 ± 17.3 kcal·kgFFM^−1^·d^−1^). Most athletes (74%) failed to meet recommendations for carbohydrate intake at home (mean 5.0 ± 1.2 g·kg^−1^·d^−1^) and 63% failed to do so at camp (mean 7.1 ± 1.6 g·kg^−1^·d^−1^). The lower threshold of the pre-exercise carbohydrate recommendations was met by 58% and 89% of athletes while percentages were 26% and 89% within 1 h after exercise, at home and at camp, respectively. None of the athletes met the recommendations within 4 h after exercise. Nutrition knowledge was associated with EA at home (r = 0.52, *p* = 0.023), and with daily carbohydrate intake at home (r = 0.62, *p* = 0.005) and at camp (r = 0.52, *p* = 0.023). Carbohydrate intake within 1 and 4 h post-exercise at home was associated with better nutrition knowledge (r = 0.65, *p* = 0.003; r = 0.53, *p* = 0.019, respectively). In conclusion, young female cross-county skiers had difficulties meeting recommendations for optimal EA and carbohydrate intake. Better nutrition knowledge may help young athletes to meet these recommendations.

## 1. Introduction

Cross-country (XC) skiing is a demanding sport, where success requires high aerobic and anaerobic capacities, as well as the ability to produce high power and speed [1,2]. To reach these requirements XC skiers periodize high amounts of training by varying exercise type, volume, intensity, and frequency between single workouts, days, weeks, and months [3,4]. The aim of the periodized training is to first overload the trainee’s physiological systems and then allow adequate recovery to develop training adaptations [5]. However, exercise-induced adaptations may be promoted or impaired by nutrition [6]. In fact, nutrition should be periodized to support the different goals of the training and to optimize competition performance [7,8]. Furthermore, XC ski training consumes high amounts of energy, which needs to be compensated for with high energy intake (EI) to maintain adequate energy availability (EA; the amount of dietary energy remaining after exercise for all other metabolic processes) and to avoid negative performance and health outcomes such as poor training response, hormonal dysfunction, impairment of bone health, and increased injury risk [9,10]. Notably, teenage female athletes may be at higher risk for stress fractures caused by low EA [11], and therefore adequate dietary intake is especially important among young female athletes. 

XC skiers compete and perform most of their key training sessions at intensities that are highly dependent on carbohydrate (CHO) based fuels for muscle metabolism [2,3,12]. Thus, pre-exercise meals should ensure adequate CHO availability in key training sessions, while this is less important before easy sessions [6,8,13]. As restoration of muscle CHO stores may take up to 24 h, the recovery process should be started as soon as possible after the high intensity training session in situations when recovery time between key training sessions is limited [14]. Meanwhile, performing part of the easy sessions with low CHO availability may increase the muscle adaptations to endurance training and therefore it might be beneficial to limit CHO intake before the training session where training intensity and quality are less important [6,8]. While CHO is an important macronutrient as a fuel, protein is needed for tissue repair and adaptation as it acts both as a trigger and substrate in anabolic processes [13,15]. Recovery and post-exercise muscle protein synthesis are optimized by ingesting adequate amounts of protein within two hours after the training session [13]. In addition to CHO and proteins, dietary fats are a vital component of the diet of XC skier as they are an important fuel during low intensity exercise and may help maintain adequate EA during hard training periods due to their high energy density [16]. Furthermore, dietary fats promote immune function and participate in the metabolism of fat-soluble vitamins [13]. Although timing macronutrient intake to support training goals is one of the key components of athletes’ nutrition, to our knowledge only one study has explored the topic among XC skiers, which demonstrated that even elite skiers had difficulties meeting adequate CHO intake around training and competition [17]. 

Optimizing nutrition is difficult without adequate knowledge. This may especially be the case among high school athletes, many of whom have moved away from their parental home and started to take more responsibility over purchasing and preparing food. Indeed, some [18,19], but not all [20], studies have shown that older athletes have better nutrition knowledge compared to younger ones. Although there is mild evidence to suggest a positive association between nutrition knowledge and dietary intake, a very limited number of studies have used valid tools to assess both nutrition knowledge and dietary intake [21]. 

Furthermore, to the best of our knowledge there are no studies reporting how nutrition knowledge is associated with timing and periodization of macronutrient intake in athletes. Therefore, the aim of the present study was to provide novel information on how CHO and protein intake were periodized around different training sessions, and then to clarify how nutrition knowledge affects dietary intake and periodization practices in young female XC skiers presenting at the top national level. In addition, we investigated how well recommendations for EA and macronutrient intake were met during different training situations.

## 2. Materials and Methods

### 2.1. Participants 

A total of 31 female XC skiers from the Finnish Ski Association’s under 18-year-old national team were invited to join the study. Of those, a total of 19 athletes (age 16.7 ± 0.7 years) participated in the study. The participants provided written informed consent prior to their involvement in the study and were allowed to drop out of the study at any time. The ethical board of the University of Jyväskylä approved the study and included procedures, and the study was conducted in accordance with the Declaration of Helsinki. 

### 2.2. Experimental Overview

The study was carried out before and during a 5-day training camp in a specific preparation season in October. Participants filled in 48-hour food and training logs before and during the training camp. Nutrition knowledge questionnaires [22] and anthropometric measurements were completed at the beginning of the training camp. 

### 2.3. Anthropometric Measurements 

Anthropometric measurements were carried out in fasted state on the first morning of the training camp. The height of the participants was measured with a stadiometer. Body mass and body composition were measured following an overnight fast using a bioimpedance (Inbody 720, Biospace Co., Seoul, Korea) measurement. 

### 2.4. Food and Training Logs

48-hour food and training logs were filled in twice during the study. The first logs were filled in between 12 and 2 days before the training camp (HOME) on self-selected days and the second logs were filled in during the second and third day of the 5-day training camp (CAMP). At CAMP, participants had three prescheduled meals in the restaurant of the local sport institute. Participants selected the contents of their meals from a buffet, which included salad and bread tables, several main course options, and dessert. Most ingredients were produced in Finland, and sport institute food is not highly processed. Typical drinks included juice from fruits, milk, and water. The energy and macronutrient contents of the dishes were obtained from the chef. Participants were allowed to have their own snacks between meals. Participants recorded the type, amount, and timing of foods and fluid consumed, measuring intake using kitchen scales. Written and verbal instructions were given for accurate record keeping. The food logs were analyzed using Aivodiet-software (version 2.0.2.3, Mashie, Malmö, Sweden), which employs the national food composition database Fineli Release 16 (2013). Energy and macronutrient intake were recorded as daily intake, as well as at pre- and post-exercise time points, and expressed in relation to body weight. 

Training logs were analyzed for exercise energy expenditure (EEE) using equations by Charlot et al. [23]. Participants’ average heart rate (HR), the duration of each training session, the subject’s body mass, self-reported maximal oxygen uptake, resting HR, and maximum HR were used for calculations. Resting energy expenditure was estimated using the Cunningham equation [24]. Resting energy expenditure that would have occurred during exercise regardless of exercise was subtracted from EEE so that only the additional energy cost of the exercise is included in the EEE. EA was estimated as EI minus EEE and expressed in kcal·kg fat-free mass (FFM)^−1^·day (d)^−1^ and compared with cut-off values for low (<30 kcal·kgFFM^−1^·d^−1^) and optimal EA (>45 kcal·kgFFM^−1^·d^−1^), as specified by Loucks et al. [10]. Sessions that involved over 30 min duration were divided into KEY or EASY sessions, as described in detail by Heikura et al. [25]. Briefly, KEY sessions were defined as speed or strength training, high intensity work (above aerobic threshold [26]) or duration of exercise over 150 min, and all other trainings were categorized as EASY sessions. The amount of ingested CHO was recorded in the 4-hour pre-exercise period as well as in 1- and 4-hour post-exercise periods, while protein intake was recorded in the 2-hour post-exercise period to compare intake with current consensus statement recommendations [13]. Mean CHO and protein intake around KEY and EASY exercises were calculated for each athlete separately at HOME and at CAMP. Thus, every athlete had single values describing her intake around different training situations (KEY at HOME; EASY at HOME; KEY at CAMP; EASY at CAMP). 

### 2.5. Nutrition Knowledge 

A validated nutrition knowledge questionnaire for young endurance athletes [22] was completed on paper at the beginning of the training camp. The questionnaire included 79 statements in true/false format, divided into five sections: (1) nutrition recommendations for endurance athletes, (2) dietary supplements, (3) fluid balance and hydration, (4) energy intake and recovery, and (5) the association between food choices and body image. Each correct answer yielded one point and each wrong answer zero points. Nutrition knowledge score refers to the proportion (percentage) of correct answers.

### 2.6. Statistical Analysis

Statistical analyses were conducted using SPSS Statistics 26 (IBM, Armonk, NY, USA). Results are reported as means ± SD. Normality was assessed via Shapiro–Wilk, and nonparametric tests were used with non-normally distributed data. Changes between HOME and CAMP as well as between different training situations were assessed using a repeated-measures analysis of variance, followed by a Student´s paired *t* test as a post hoc test to determine the *p* values in the pairwise comparisons. Wilcoxon signed rank test was used in the case of non-normally distributed variables. Effect sizes were calculated as Cohen’s *d* with threshold values of <0.2 (trivial), 0.2–0.5 (small), 0.5–0.8 (moderate), and >0.8 (large) [27]. Pearson’s (normally distributed data) or Spearman’s (non-normally distributed data) correlation coefficient was used to analyze correlations between nutrition knowledge score and other variables. To test knowledge about nutrition recommendations more specifically, part one (44 questions) of the 79 question nutrition knowledge questionnaire [22] was used in the correlation analysis. Statistical significance was defined as *p* < 0.05. 

## 3. Results

### 3.1. Dietary Intake

Table 1 shows daily EI, EEE, EA, and macronutrient intake at HOME and at CAMP. Daily intake of energy (*p* < 0.001, d = 1.69), protein (*p* = 0.002, d = 0.84), and CHO (*p* < 0.001, d = 2.36), as well as EEE (*p* < 0.001, d = 2.21) were lower at HOME compared to CAMP, while an increased trend from HOME to CAMP was observed in EA (*p* = 0.065, d = 0.47). A high percentage, 89% and 58% of athletes, had suboptimal EA at HOME and at CAMP, respectively. Furthermore, five (26%) athletes had low EA at HOME and seven (37%) athletes at CAMP.

EEE and macronutrient intake around KEY and EASY sessions are presented in Table 2. EEE was significantly higher in KEY sessions compared to EASY sessions at HOME and at CAMP (*p* = 0.007, d = 0.72; *p* < 0.001, d = 1.64, respectively). Furthermore, EEE in KEY sessions was lower at HOME than at CAMP (*p* < 0.001; d = 1.14). CHO intake during the 4-hour period before KEY sessions was lower at HOME than at CAMP (*p* = 0.002, d = 0.74), while CHO intake before EASY sessions remained similar (*p* = 0.37, d = 0.28). CHO intake both in 1 and 4 hours after exercise period was significantly lower at HOME compared to CAMP after KEY (*p* < 0.001, d = 1.17; *p* < 0.001; d = 1.40, respectively) and EASY (*p* = 0.002, d = 1.04; *p* < 0.001; d = 1.09, respectively) sessions. Protein intake was higher after KEY sessions at CAMP compared to EASY sessions at CAMP (*p* = 0.033, d = 0.41) and KEY sessions at HOME (*p* = 0.018, d = 0.59). Many athletes failed to meet the lower end of the recommended CHO intake on a daily basis as well as around KEY exercises while the percentages were higher at HOME compared to CAMP. All athletes met the minimum recommendations for protein intake. 

### 3.2. Nutrition Knowledge

Mean total nutrition knowledge score was 76.0 ± 7.3%. The scores from the different parts were 73.4 ± 8.4% (Part 1), 67.4 ± 19.1% (Part 2), 92.5 ± 10.0% (Part 3), 71.8 ± 14.6% (Part 4), and 87.1 ± 11.3% (Part 5). 

### 3.3. Nutrition Knowledge, Exercise Energy Expenditure and Dietary Intake 

**Figure 1:**
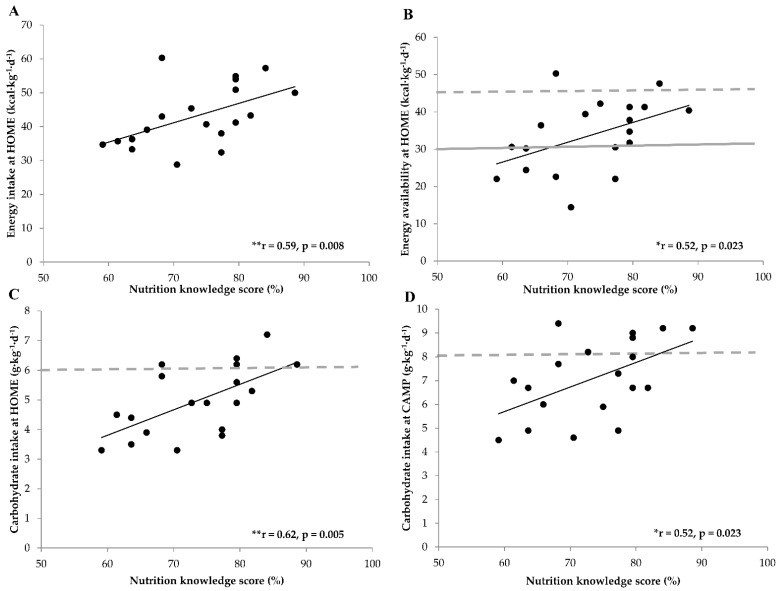
Associations between nutrition knowledge score and daily energy intake (**A**), energy availability (**B**), and carbohydrate intake (**C**,**D**). The gray horizontal dotted line denotes the consensus statement recommendations for optimal energy availability and carbohydrate intake [10,13]. The gray horizontal line denotes the threshold for low energy availability [10]. HOME = during home training; CAMP = during training camp; * *p* < 0.05; ** *p* < 0.01 significant correlation.

Figure 1 demonstrates that a higher nutrition knowledge score was associated with higher EI, EA, and CHO intake at HOME (r = 0.59, *p* = 0.008; r = 0.52, *p* = 0.023, r = 0.62, *p* = 0.005, respectively), as well as with higher CHO intake at CAMP (r = 0.52, *p* = 0.023). EI, EA, and protein intake at CAMP were positively associated with nutrition knowledge, but correlations were not statistically significant (r = 0.44; *p* = 0.062; r = 0.32; *p* = 0.19; r = 0.44, *p* = 0.063, respectively). Daily EEE did not correlate with nutrition knowledge at HOME (r = 0.22; *p* = 0.36) or at CAMP (r = 0.12; *p* = 0.62). 

### 3.4. Nutrition Knowledge, Exercise Energy Expenditure and Dietary Intake 

**Figure 2:**
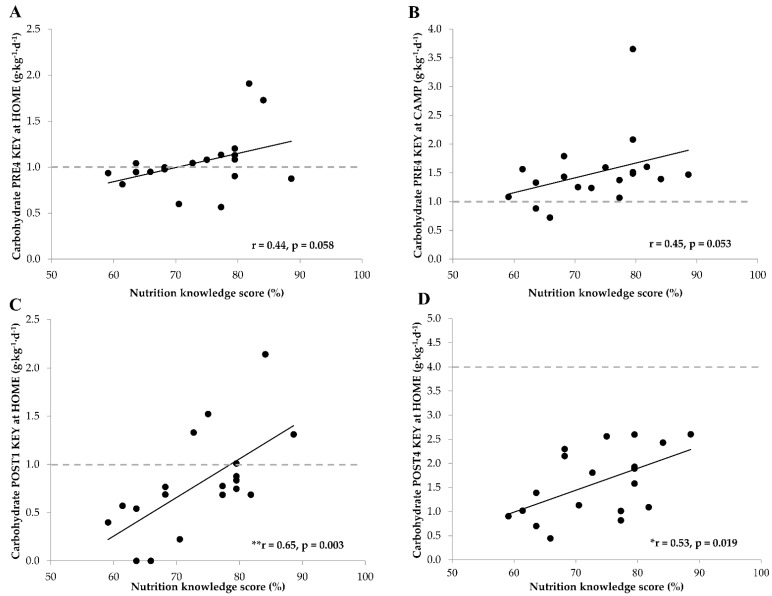
Associations between nutrition knowledge score and carbohydrate intake before (**A**,**B**) and after (**C**,**D**) KEY training sessions. The gray horizontal dotted line denotes the consensus statement recommendations for optimal carbohydrate intake [13]. PRE4 = within four hours before exercise; POST1 = within an hour after exercise; POST4 = within four hours after exercise; HOME = during home training; CAMP = during training camp; KEY = key training session; * *p* < 0.05; ** *p* < 0.01 significant correlation.

There was a positive trend between nutrition knowledge score and CHO intake before KEY exercises at HOME (r = 0.44, *p* = 0.058, Figure 2A) and at CAMP (r = 0.45, *p* = 0.053, Figure 2B). In addition, nutrition knowledge score was positively associated with CHO intake during 1-hour (r = 0.65, *p* = 0.003, Figure 2C) and 4-hour (r = 0.53, *p* = 0.019, Figure 2D) periods after KEY sessions at HOME but not at CAMP (r = −0.01, *p* = 0.97; r = 0.16; *p* = 0.49, respectively). Post-exercise protein intake and EEE during KEY exercises were not associated with nutrition knowledge at HOME (r = −0.08, *p* = 0.76; r = 0.04, *p* = 0.86, respectively) or at CAMP (r = 0.33, *p* = 0.18; r = 0.17, *p* = 0.49, respectively).

## 4. Discussion

The main findings of this study were that nutrition knowledge was positively associated with (i) daily EA, (ii) daily CHO intake, and (iii) CHO intake around exercise. It is notable that young female XC skiers had significant difficulties meeting the recommended CHO intake on a daily basis as well as during the 4-hour pre- and post-exercise periods. Detected difficulties may be limited with adequate nutrition knowledge and with arrangements that reduce an athlete’s own responsibility for planning, scheduling, and preparing their own meals. It was also observed that young female skiers periodized their energy and macronutrient intake according to the energy requirements set by different EEE between training periods while dietary intake remained approximately similar before and after single training sessions with various intensities and durations.

The results demonstrated that young female XC skiers had suboptimal EA and CHO intake, especially during their typical home training. According to our observations, participants had about double the EEE during the CAMP compared to typical training at HOME as well as in KEY sessions compared to EASY sessions, which highlights the higher energy and macronutrient needs in these situations. Athletes increased their dietary intake from HOME to CAMP and therefore periodized their nutrition between normal and intensified training periods. In fact, the percentage of athletes who met the optimal level of EA and CHO intake was higher at CAMP than at HOME, which suggests that most athletes adjusted their diet to better support their performance and recovery during high load training camp. The scheduled and prepared buffet meals may also have supported athletes in meeting their energy and macronutrient requirements during the CAMP. Nevertheless, most athletes had inadequate CHO intake in both conditions despite dietary adjustments, which is a common observation among endurance athletes [17,25,28]. These findings are concerning, as inadequate CHO intake may lead to decreased glycogen stores that may impair training intensity and quality during intensive training [12,29] and increase the risk of illness [30].

Differences in periodization practices between EASY and KEY sessions were minor, and even the small differences that did occur (higher CHO intake before EASY sessions KEY sessions) actually goes against the principle of ‘fuel for the work required’ [31]. Indeed, almost half the athletes failed to meet pre-exercise CHO recommendations before KEY training sessions at HOME suggesting that training quality may be continuously compromised by fuel deficiencies. Another concerning finding was that 74% of athletes did not consume sufficient CHO during the first hour after KEY exercises at HOME, and almost all the athletes failed to meet recommendations within 4 hours post-exercise both at HOME and at CAMP. As restoration of muscle CHO stores may take up to 24 h, insufficient post-exercise CHO intake may have been particularly harmful at CAMP where the time between subsequent KEY sessions was limited [14]. It is not uncommon for female endurance athletes to have difficulties meeting post-exercise CHO recommendations, as only 40% of elite endurance runners met the recommendation within 1 hour of exersice [25] and only 31–54% of elite skiers did so within 4 hours of exercise [17]. Meanwhile, pre-exercise targets have been reported being met by 74–100% of elite female endurance athletes [17,25], which is a similar proportion to that observed in our participants at CAMP (89%) but higher than at HOME (58%). 

The nutrition knowledge detected in this study was similar to previous studies that have used the same questionnaire for young endurance athletes [19,20]. As a novel finding, we detected that higher nutrition knowledge was associated with higher EA and CHO intake. When considering that 58–89% of athletes had suboptimal EA and 26–37% had low EA, the findings of this study highlight the importance of adequate nutrition knowledge to prevent the negative health and performance consequences associated with inadequate EA [9]. Furthermore, daily CHO intake was at a suboptimal level in 63–74% of athletes, which suggests that better nutrition knowledge may help to maintain adequate fueling for optimal performance and recovery [13]. 

In addition to daily intake, nutrition knowledge may significantly support recommended CHO intake around KEY training sessions. In the present study, athletes with higher nutrition knowledge were more likely to have higher CHO intake in pre- and post-exercise periods. This observation was pronounced at HOME, where athletes have more responsibility over the contents and timing of their meals, suggesting that enhanced nutritional education may be needed for athletes who have recently moved away from their parental home. Most athletes in this study belonged to sport high schools, which enabled them to have a higher training volume that they enjoyed in their earlier years in secondary school. However, inadequate post-exercise CHO intake may delay recovery from an increased training load [14,29], which may increase the risk of overtraining [32]. Suboptimal pre-exercise CHO intake, in turn, may decrease the quality of KEY training sessions [8,29,31], diminishing the desirable performance outcomes. Therefore, it is essential that athletes have adequate levels of nutrition knowledge to allow sufficient fueling around training sessions as well as adequate daily energy intake and availability to support training adaptations and overall health.

We found that young female XC skiers had slightly higher daily and more than twofold post-exercise protein intake compared to recommendations regardless of training type and nutrition knowledge. However, even higher protein intakes have been observed in elite female XC-skiers [17]. According to the current consensus statements, athletes have been recommended to consume 1.2–2.0 g·kg^−1^·d^−1^ and 0.25–0.30 g·kg^−1^ of protein during the 2-hour post-exercise period [13]. A recent investigation by Churchward-Venne et al. [33] found that 0.49 g·kg^−1^ protein is needed to maximize protein synthesis rate after 90 min of cycling at ∼60% VO_2_peak, which suggests that ~50% higher protein intake may be needed after endurance workouts than earlier detected in the context of strength training [34]. Furthermore, Macnaughton et al. [35] found that a higher than currently recommended amount of protein is needed to maximize protein synthesis after a whole-body workout. In the case of our subjects, energy deficiency [36] and low CHO availability [37] may have further increased the requirements. While no studies exist specific to XC skiing, it is not baseless to suggest that protein requirements may be over current consensus statements for athletes who use their whole-body musculature during high load endurance training. It remains to be investigated whether as high as ~0.8 g·kg^−1^ post-exercise protein intake, which was detected in this study, has any benefits on recovery and adaptation.

Fat intake was observed to be ∼30% of daily EI at HOME and ∼32% at CAMP. Therefore, fat intake was within the recommended range of 20–35% of daily EI [13]. Adequate fat intake may have been an important component in supporting the health status of the athletes and may have aided in avoiding a more severe energy deficit [16]. These findings, in combination with previous studies [17], suggest that achieving the recommended level of fat intake is not a major challenge for XC skiers. 

### Limitations 

Self-reported food logs may cause bias due to misreporting [38], and rather inaccurate methods used to assess EEE [23] and FFM, as well as the short recording period, may have further distorted EA assessment. Nevertheless, weighted food logs and highly motivated participants may have reduced bias related to misreporting. An additional limitation was that exercises were roughly divided into KEY and EASY sessions, and the exact loads of the exercises may have varied between subjects. However, EEE was significantly higher at KEY sessions compared to EASY sessions, and was not associated with nutrition knowledge. Finally, our research team took great care to continually interact with the participants to encourage their full compliance with the study protocols.

## 5. Conclusions

We found that young female XC skiers had suboptimal CHO intake and timing, both on a daily basis as well as around exercises, while protein intake was significantly higher than currently recommended [13]. Athletes periodized their dietary intake between training periods with different loads, as energy and macronutrient intake increased from normal training to an intensified training camp. Nevertheless, many athletes had suboptimal EA and CHO intake in both conditions that may have unfavorable performance and health outcomes. As a novel finding, we detected that adequate nutrition knowledge may help young athletes to meet recommended levels of EA and CHO intake. Thus, our findings highlight the importance of enhanced high-quality nutrition education for young endurance athletes. 

## Figures and Tables

**Table 1:** Mean (± SD) daily training volume, energy and macronutrient intake, exercise energy expenditure, and energy availability in young female cross-country skiers (n = 19).

	HOME	%	CAMP	%	Recommendation [10,13]
Training (min·d^−1^)	120 ± 26	NA	214 ± 20 ***	NA	NA
EI (kcal·kg^−1^·d^−1^)	43.1 ± 9.1	NA	60.4 ± 13.1 ***	NA	NA
EEE (kcal·kg^−1^·d^−1^)	14.9 ± 4.5	NA	26.8 ± 4.3 ***	NA	NA
EA (kcal·kg^−1^·d^−1^)	33.7 ± 9.6	11	40.3 ± 17.3	42	≥45
Protein (g·kg^−1^·d^−1^)	2.1 ± 0.3	100	2.5 ± 0.5 **	100	1.2–2.0
CHO (g·kg^−1^·d^−1^)	5.0 ± 1.2	26	7.1 ± 1.6 ***	37	6–10/8–12
Fat (% of EI)	30 ± 6	95	32 ± 7	100	20–35

EI = energy intake; EEE = exercise energy expenditure; EA = energy availability; CHO = carbohydrate; HOME = during home training; CAMP = during training camp; % = the percentage of athletes who met the lower threshold for recommended intake for optimal performance and recovery [10,13]; NA = Not Applicable. ** *p* < 0.01; *** *p* < 0.001 significant difference between HOME and CAMP.

**Table 2:** Mean (± SD) daily training volume, energy and macronutrient intake, exercise energy expenditure, and energy availability in young female cross-country skiers (n = 19).

	HOME			CAMP			Recommendation [13]
	EASY	KEY	%	EASY	KEY	%	
EEE (kcal·kg^−1^·d^−1^)	7.3 ± 3.5	10.8 ± 3.6 ^††^	NA	8.3 ± 3.3	14.6 ± 1.8 ***^,^^†††^	NA	NA
Protein POST2 (g·kg^−1^)	0.6 ± 0.3	0.6 ± 0.2	100	0.7 ± 0.3	0.8 ± 0.1 *^,^^†^	100	0.25–0.30
CHO PRE4 (g·kg^−1^)	1.2 ± 0.8	1.0 ± 0.3	58	1.7 ± 1.2	1.3 ± 0.3 **	89	1–4
CHO POST1 (g·kg^−1^)	0.5 ± 0.5	0.8 ± 0.5	26	1.7 ± 1.0 ***	1.7 ± 0.7 ***	89	1.0–1.2
CHO POST4 (g·kg^−1^)	1.5 ± 0.7	1.6 ± 0.7	0	2.7 ± 1.2 ***	2.7 ± 0.8 ***	5	4.0–4.8

EEE = exercise energy expenditure; CHO = carbohydrate; PRE4 = within four hours before exercise; POST1 = within an hour after exercise; POST2 = within two hours after exercise; POST4 = within four hours after exercise; HOME = during home training; CAMP = during training camp; KEY = key training sessions; EASY = non-KEY training sessions; % = the percentage of athletes who met the lower threshold for recommended intake for optimal performance and recovery [13] around key sessions; NA = Not Applicable. * *p* < 0.05; ** *p* < 0.01; *** *p* < 0.001 significant difference between HOME and CAMP; ^†^ *p* < 0.05; ^††^ *p < 0.01;* ^†††^ *p* < 0.001 significant difference between EASY and KEY.

## Data Availability

The data presented in this study are available upon reasonable request from the corresponding author.
